# The Effect of PDMS Incorporation on the Physicochemical Properties of Acrylate-Based Resins for SLA-Based 3D Printing

**DOI:** 10.3390/polym18070827

**Published:** 2026-03-28

**Authors:** Yura Choi, Jayoung Hyeon, Jinyoung Kim, Eunsu Park, Namchul Cho

**Affiliations:** Department of Energy Engineering, Soonchunhyang University, Asan 31538, Republic of Korea; bnbn3238@sch.ac.kr (Y.C.);

**Keywords:** 3D printing, polydimethylsiloxane, stereolithography, acrylate PDMS, photo-curable polymer

## Abstract

A photo-curable silicone-modified resin system based on polydimethylsiloxane (PDMS) was developed and systematically evaluated for stereolithography (SLA)-based 3D printing applications. The resin formulation consisted of bisphenol A ethoxylate dimethacrylate (Bis-EMA) and trimethylolpropane triacrylate (TMPTMA) as reactive monomers, with methacrylate-terminated PDMS (PDMS-MMA) incorporated at concentrations ranging from 0 to 15 wt%. The influence of PDMS-MMA content on key physicochemical properties relevant to SLA processing, including viscosity, mechanical performance, thermal stability, optical transmittance, and curing shrinkage, was systematically investigated. Moderate incorporation of PDMS-MMA improved the mechanical flexibility of the resin, with the tensile strength reaching a maximum value of 5.95 MPa at 5 wt% PDMS-MMA. However, further increases in PDMS-MMA content resulted in a gradual decrease in tensile strength and optical transmittance, indicating the importance of optimizing the formulation composition. Thermogravimetric analysis (TGA) indicated improved thermal stability with increasing PDMS-MMA content, while curing shrinkage decreased progressively as the PDMS fraction increased. Structural printing tests confirmed that the developed resin system exhibited stable layer adhesion and shape fidelity during SLA fabrication, enabling the successful printing of complex three-dimensional structures. These results demonstrate that PDMS-modified acrylate resins provide a promising strategy for balancing mechanical flexibility, dimensional stability, and printability in SLA-based additive manufacturing.

## 1. Introduction

Liquid photosensitive resins are employed in ultraviolet (UV)-curable three-dimensional (3D) printing, which serves as a core technology for rapid prototyping [1,2]. As a representative photopolymerization process, this technique harnesses the precise control of UV light to activate crosslinking reactions in photo-reactive polymers, with acrylate- and epoxy-derived resins constituting the most widely used material platforms [3]. Among various additive manufacturing methods, stereolithography (SLA) provides the highest resolution currently achievable, utilizing UV light to initiate the photopolymerization of liquid photo-reactive resins. This technology employs a layer-by-layer photopolymerization process, wherein individual layers of monomeric resin are sequentially solidified, thereby enabling the fabrication of finely resolved microscale structures [4,5]. This sequential curing mechanism facilitates the fabrication of micron-scale features characterized by high surface quality and superior manufacturing efficiency. In addition to its high dimensional precision and superior surface finish, the method is further distinguished by its energy efficiency and consistent manufacturing reliability [6,7]. The ability to fabricate highly intricate and geometrically complex components underscores a fundamental advantage over conventional manufacturing techniques, which lack the precision and flexibility necessary for producing such sophisticated structures [4,6,7,8]. However, despite these advantages, SLA resins often suffer from volumetric shrinkage during polymerization, leading to compromised dimensional accuracy and reduced mechanical strength. Addressing this polymerization-induced shrinkage while preserving dimensional fidelity and optical transparency remains a key challenge in developing next-generation SLA photopolymer resins [9,10,11,12]. Silicone elastomers have emerged as highly attractive materials for a wide range of technological applications. Their versatility stems from a distinct combination of intrinsic properties, including exceptional elasticity, high resilience, excellent thermal insulation, and favorable biocompatibility [13]. Silicone has found extensive utility across numerous applications, ranging from domestic commodities and sealants to energy-dissipating materials, flexible electronics, microfluidic systems, and biomedical devices [14,15]. Conventionally, the industrial fabrication of silicone materials relies on thermosetting crosslinking mechanisms such as platinum-catalyzed hydrosilylation, condensation polymerization, and peroxide-initiated radical curing. These processes typically involve molding techniques, such as injection molding, to produce functional components. These processes typically entail molding the resulting materials into functional components using specialized injection molding methods. However, such approaches are often unsuitable for rapid or customized production owing to the high cost and limited adaptability of mold-based systems [16,17].

Polydimethylsiloxane (PDMS)-based silicone resins have attracted considerable attention owing to their outstanding thermal stability, chemical inertness, and mechanical flexibility [6,18,19]. Traditional PDMS systems, which cure through thermal or two-part mixing processes, are inherently unsuitable for integration with UV-curable 3D printing platforms [20,21]. Recent advances in silicone chemistry have facilitated the development of UV-responsive systems based on several distinct mechanisms, including radical photopolymerization in acrylate-functional silicones, thiolene reactions in thiol-functional silicones, and cationic curing in epoxy-functional silicones [22,23]. Among these, acrylate-functional silicones present multiple advantages for additive manufacturing, including high polymerization efficiency, tunable crosslink density, resistance to oxygen inhibition, cost-effectiveness, and ease of formulation. These features establish acrylate–PDMS hybrids as highly promising candidates for UV-based 3D printing of soft and flexible structures [24,25,26,27].

Acrylate-functional silicones possess several notable advantageous features, including rapid polymerization kinetics, tunable structural design, cost-effectiveness, ease of processing, and resistance to oxygen inhibition, which collectively make them highly promising candidates for UV-cured 3D printing applications [25,28,29,30]. Despite their advantages, acrylate-functional silicones are frequently associated with several limitations, the most significant of which is excessive volumetric shrinkage during curing. Such shrinkage often leads to considerable dimensional inaccuracies in the printed structures, thereby hindering their ability to meet precise design requirements [31]. This limitation becomes especially critical in applications requiring high-precision components, such as microfluidic devices, optical lenses, and complex mold structures. The transparency of additively manufactured silicone structures is particularly important in optical fields, as clear and distortion-free materials are essential for ensuring reliable light transmission and high-fidelity imaging [32]. Numerous UV-curable silicone resins fail to achieve adequate transparency and frequently undergo a decline in mechanical properties upon curing, which consequently reduces their effectiveness in advanced optical and engineering applications [33,34,35].

Several studies have reported the development of silicone or polysiloxane-modified photo-curable resins for additive manufacturing applications. For example, low-shrinkage transparent UV-cured silicone resins for 3D printing were developed by incorporating silicone components into photo-curable systems, demonstrating improved dimensional stability and optical properties [6]. Similarly, hyperbranched polysiloxane-modified light-curable resins that exhibited enhanced toughness and improved mechanical performance [27]. 

Despite these advances, systematic investigations into the composition–property relationships of PDMS-modified acrylate resin systems remain limited. In particular, the influence of PDMS concentration on viscosity, mechanical flexibility, thermal stability, optical transmittance, and curing shrinkage has not been comprehensively clarified. Although significant progress has been made in the development of silicone-modified photo-curable resins for additive manufacturing, several technical challenges remain in acrylate-based SLA resin systems. In particular, the incorporation of silicone components into acrylate-based systems can introduce phase compatibility issues due to the intrinsic polarity difference between siloxane segments and acrylate matrices. Such heterogeneity may influence the viscosity, curing behavior, and optical transparency of the resin system. In addition, excessive silicone incorporation may reduce the effective crosslink density of the cured network, potentially leading to deterioration of mechanical strength [6,27].

Compared with other modification approaches, such as nanoparticle reinforcement or elastomer blending, PDMS-based modification offers several advantages including high chain flexibility, low surface energy, and excellent thermal stability. These characteristics make PDMS an attractive candidate for reducing curing shrinkage and improving the flexibility of SLA photo-curable resins while maintaining good processability during printing.

In this study, the influence of PDMS-MMA incorporation on the physicochemical properties of a photo-curable acrylate resin system was systematically investigated. The resin formulation consisted of bisphenol A ethoxylate dimethacrylate (Bis-EMA), trimethylolpropane triacrylate (TMPTMA), and diphenyl (2,4,6-trimethylbenzoyl) phosphine oxide (TPO) as the photoinitiator. PDMS-MMA was incorporated at concentrations ranging from 0 to 15 wt% to evaluate its effects on key parameters relevant to SLA processing, including resin viscosity, curing shrinkage, mechanical properties, optical transmittance, and thermal stability. Test specimens were fabricated using SLA-compatible 3D printing to simulate practical processing conditions. By systematically analyzing the composition–property relationships of PDMS-modified acrylate resins, this study provides insights into the design and optimization of silicone-modified photopolymers for additive manufacturing applications.

## 2. Materials and Methods

### 2.1. Materials

Bis-EMA and TMPTMA were obtained from Miwon Chemical Co., Ltd. (Anyang, Republic of Korea). TPO served as the photoinitiator, effectively absorbing UV light at 405 nm to initiate the polymerization process. It was purchased from Miwon Chemical Co., Ltd. (Anyang, Republic of Korea). The bi-functional aliphatic urethane acrylate oligomer and α,ω-dimethacrylate-terminated polydimethylsiloxane (AS-PDMS-M; Mn = 1300–1400 g/mol) were purchased from Asan Materials Co., Ltd. (Asan, Republic of Korea). Sodium dodecyl sulfate (SDS) was obtained from Junsai Co., Ltd. (Tokyo, Japan), and all other reagents were purchased from commercial suppliers and used without further purification.

### 2.2. Preparation of Clear Photopolymer Base Resin

The base photo-curable resin formulation used for SLA-based 3D printing was prepared by blending Bis-EMA, TMPTMA, and a bi-functional urethane acrylate oligomer at a weight ratio of 8.0:1.5:0.5. A TPO-based photoinitiator was added at 4.5 wt% relative to the total resin weight to ensure efficient photoinitiation during curing. The mixture was dispersed via ball milling at 200 rpm for 12 h at 25 ± 1 °C under light-shielded conditions to prevent unintended photopolymerization. After milling, the resin was degassed under vacuum at ambient temperature to remove trapped air bubbles prior to use.

PDMS-MMA was incorporated into the prepared photo-curable base resin at weight fractions of 0–15 wt%, as detailed in Table 1. The resulting mixtures were stirred at 150 rpm for 10 h at room temperature to ensure homogeneous dispersion. To suppress phase separation due to the low polarity of PDMS-MMA, SDS was used as an anionic surfactant. The amount of SDS was adjusted according to the PDMS content, varying between 0.1 and 1.5 wt% of the total resin weight. After stirring, undispersed surfactant aggregates were removed by filtration using stainless steel mesh screens (100–150 mesh) to eliminate large particulates and ensure homogeneous dispersion of the resin blend. The final resin blends were subsequently used for specimen fabrication.

### 2.3. SLA Printing Conditions

Three-dimensional printing was performed using an A+ SLA 3D printer (Shindoh Co., Ltd., Seoul, Republic of Korea) equipped with a 405 nm laser diode (nominal output power: 600 mW).

The layer thickness was set to 50 μm, and all samples were printed under identical processing parameters to ensure consistent comparison between resin formulations. The printed specimens were oriented horizontally on the build platform to minimize structural distortion during fabrication.

After printing, the specimens were rinsed with ethanol to remove uncured resin and subsequently post-cured under a UV curing system (405 nm, 80 W) for approximately 5 min at room temperature to ensure complete polymerization of the printed structures [36].

### 2.4. Analysis of Viscosity

The viscosity of each photo-curable resin was measured using a rotational viscometer (DV2TLV Rheometer, Brookfield, Middleboro, MA, USA) at a controlled temperature of 24 ± 1 °C. Viscosity measurements were performed at a constant rotation speed of 60 rpm for 2 min, and the results were recorded in centipoise (cP). Each formulation was assessed in triplicate, and the mean value was reported.

### 2.5. Analysis of Tensile Strength

Mechanical tensile testing was conducted using a universal testing machine (AGS-X, Shimadzu Corporation, Kyoto, Japan) equipped with a 10 kN load cell, in accordance with the ASTM D638 [37] standard (Type V specimens). The 3D-printed dog-bone specimens had dimensions of 63 mm (length) × 5 mm (width) × 2 mm (thickness), with a gauge length of 10 mm. Tests were performed at a constant crosshead speed of 10 mm/min. Each formulation was tested using at least three independent specimens, and the reported tensile strength and elongation values represent the average results. The corresponding standard deviations were calculated and included to evaluate the experimental variability.

### 2.6. Analysis of Thermal Properties

The thermal properties of the PDMS-MMA resins were evaluated using thermos-gravimetric analysis (TGA). TGA was performed using a thermos-gravimetric analyzer (Q50, TA Instruments, New Castle, DE, USA) in which approximately 10–15 mg of each cured sample was heated from room temperature to 700 °C at a heating rate of 10 °C/min under a nitrogen flow atmosphere. The onset decomposition temperature (T_5_%), the temperature corresponding to the maximum weight loss rate (Tₘₐₓ), and the residual mass at 700 °C were recorded. Each measurement was conducted in triplicate, and the reported values represent the average results.

### 2.7. Analysis of Optical Transmittance

Optical transmittance was measured using a UV–Vis spectrophotometer (Fluoro-Max-4, HORIBA Scientific, Kyoto, Japan) over the wavelength range of 200–800 nm. The samples were fabricated as flat sheets with dimensions of 1 cm (width) × 3 cm (length) × 4 mm (thickness). After 3D printing, residual uncured resin was removed from the surface using ethanol, and UV post-curing was carried out for 30 s. All measurements were conducted at room temperature. All optical transmittance measurements were performed using three independent samples for each formulation, and the reported values represent the average results.

### 2.8. Analysis of Shrinkage

Linear and volumetric shrinkage were evaluated using square specimens (20 mm × 20 mm) fabricated by SLA 3D printing under identical processing conditions. The designed side length (L_0_ = 20 mm) was compared with the final dimension (L) measured after printing and UV post-curing. Dimensional measurements were carried out using a digital caliper with an accuracy of ±0.01 mm.

Linear shrinkage (%) was calculated using the following equation:

Linear shrinkage (%) = (L_0_ − L)/L_0_ × 100 (1)

Volumetric shrinkage (%) was estimated assuming isotropic contraction, according to the following:

Volumetric shrinkage (%) = (V_0_ − V)/V_0_ × 100 (2)

where V_0_ and V represent the initial and final volumes of the specimen, respectively. All measurements were performed in triplicate and averaged.

## 3. Results and Discussion

**Figure 1:**
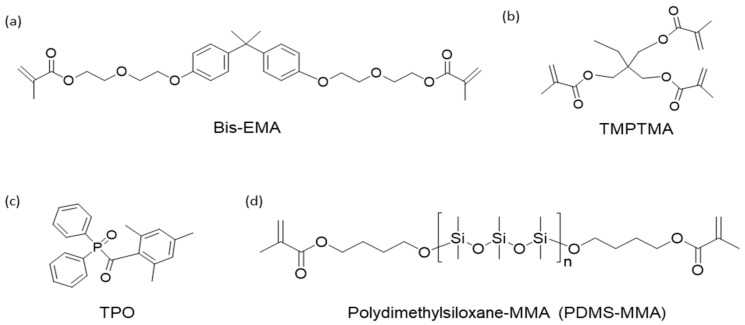
Chemical structures of the materials: (**a**) Bis-EMA, (**b**) TMPTMA, (**c**) the initiator (TPO), and (**d**) methacrylate-terminated PDMS-MMA (AS-PDMS-M; Mn = 1300–1400 g/mol).

In this study, photopolymers were prepared by mixing acrylate monomers (Bis-EMA), a bi-functional aliphatic urethane acrylate oligomer, and a crosslinker (TMPTMA) (Figure 1).

### 3.1. Viscosity of Organic PDMS-MMA Resins

**Figure 2:**
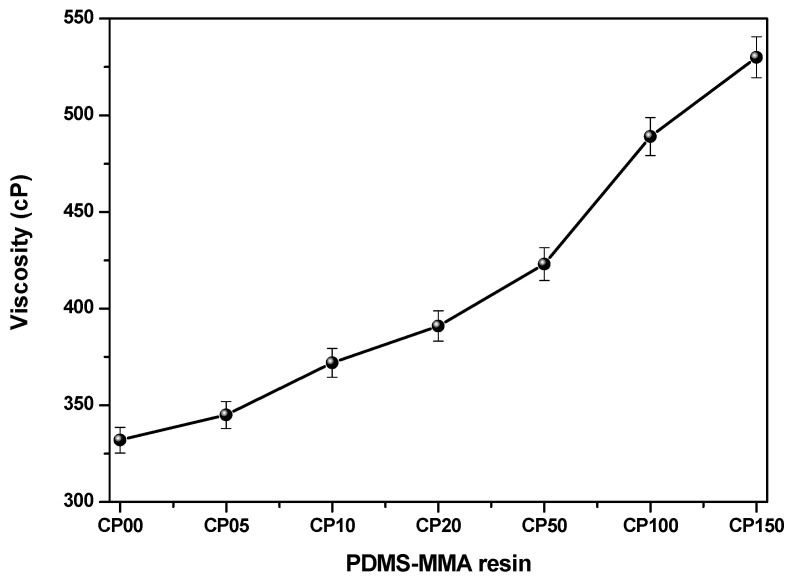
Viscosity values for polydimethylsiloxane-MMA (PDMS-MMA). Error bars represent standard deviation (*n* = 3).

The incorporation of PDMS-MMA into the acrylate-based photo-curable resin markedly influenced the viscosity of the resulting formulation. As shown in Table 2 and Figure 2, the viscosity of the base resin (CP0) was approximately 332 cP, whereas that of the formulation containing 15 wt% PDMS-MMA (CP150) increased to 530 cP at 24 ± 1 °C.

PDMS-MMA, being a linear silicone polymer, exhibits a high degree of chain flexibility and segmental mobility, which can contribute substantially to viscosity even at low concentrations. Furthermore, the partial phase incompatibility between PDMS and acrylate matrix promotes micro-domain formation, thereby increasing internal friction under shear flow.

The incorporation of PDMS-MMA increased the viscosity of the resin formulations. This trend indicates that the addition of PDMS-MMA influences the rheological behavior of the acrylate-based resin system. SDS was introduced to improve the dispersion stability of PDMS-MMA within the resin matrix prior to printing. The viscosity increased monotonically with PDMS-MMA content, indicating consistent processing behavior across the formulations. While moderate viscosity is beneficial for maintaining structural integrity during printing, excessive viscosity may hinder resin recoating and interlayer adhesion. In this study, a PDMS-MMA concentration range of 5–15 wt% provided a suitable balance between printability and material performance.

The increase in viscosity with increasing PDMS-MMA content may also be associated with the partial incompatibility between silicone segments and the acrylate-based matrix. Previous studies have reported that silicone-modified photo-curable resin systems can exhibit microphase-separated structures due to the intrinsic polarity difference between siloxane chains and acrylate networks. Such microstructural heterogeneity may increase flow resistance and lead to higher apparent viscosity during shear deformation.

In the present study, the viscosity increase observed with increasing PDMS-MMA concentration is consistent with these previously reported trends in silicone-modified acrylate systems. Although direct microscopic morphology characterization (e.g., SEM or AFM) was not performed in this work, the rheological behavior suggests that the incorporation of PDMS-MMA may influence the microstructural organization of the resin matrix [3,4,6]. Further investigation using microscopic techniques would provide additional insight into the dispersion state and phase structure of PDMS domains within the cured network.

### 3.2. Tensile Properties of PDMS-MMA Resins

**Figure 3:**
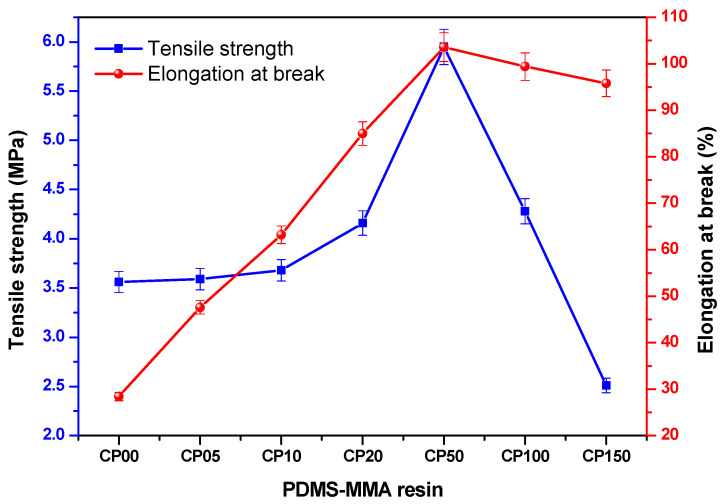
Tensile properties of the PDMS-MMA resins. Error bars represent standard deviation (*n* = 3).

In this study, uniaxial tensile tests were conducted to evaluate the mechanical properties of acrylate-based photo-curable resins containing varying concentrations of PDMS-MMA. Both tensile strength and elongation at break exhibited systematic variation as a function of PDMS-MMA content (Table 2; Figure 3).

The tensile strength of the unmodified (PDMS-free) resin (CP0) was approximately 3.56 MPa. As the PDMS-MMA content increased from 0 to 5 wt%, the tensile strength increased, reaching a maximum of 5.95 MPa at CP50. However, at higher concentrations (10–15 wt%), the tensile strength deceased, with the CP150 exhibiting a lower value of 2.51 MPa. This trend suggests that moderate PDMS-MMA incorporation can improve mechanical performance, whereas excessive incorporation may interfere with effective network formation.

A similar trend was observed for elongation at break. The elongation increased up to 5 wt% PDMS-MMA, reaching a maximum of 103.6% for CP50 specimen. This enhancement can be attributed to the incorporation of flexible siloxane segments, which increase chain mobility and allow for greater deformation under tensile loading. However, at higher loadings (10–15 wt%), elongation decreased slightly. This reduction may be associated with increased microstructural heterogeneity arising from the polarity difference between PDMS segments and the acrylate matrix at elevated PDMS contents, which may reduce network uniformity and affect stress transfer efficiency within the cured matrix. Consequently, an optimal PDMS-MMA concentration appears necessary to balance flexibility and structural integrity.

Due to the limited availability of reliable compressive strength data, this study primarily focused on tensile-derived mechanical properties. Further mechanical characterization, including compressive and flexural testing, will be explored in future work to provide a more comprehensive understanding of the resin’s mechanical behavior.

### 3.3. Thermal Properties of PDMS-MMA Resins

**Figure 4:**
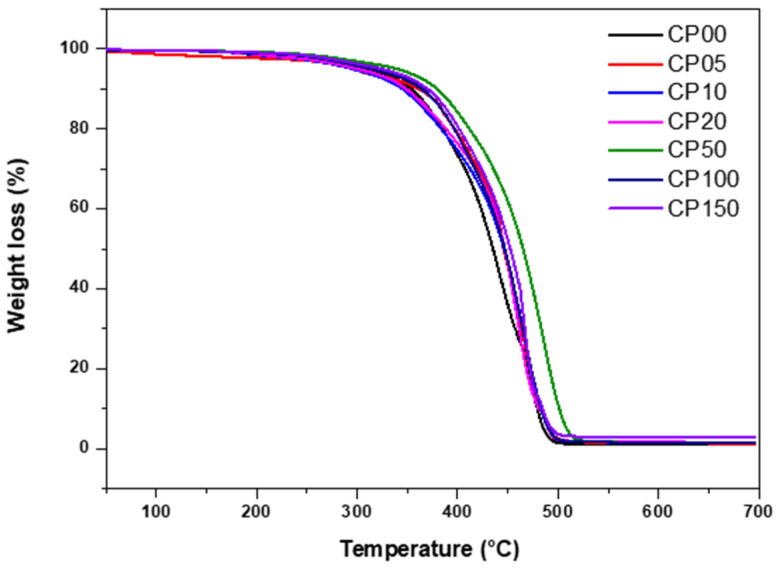
TGA curves of different PDMS-MMA resins.

TGA was conducted to assess the thermal stability of photo-curable acrylate-based resins containing various concentrations of PDMS-MMA. Each cured resin specimen was heated from room temperature to 700 °C at a constant heating rate of 10 °C/min under a nitrogen purge atmosphere. During the analysis, the onset decomposition temperature (T_5_%), the temperature corresponding to the maximum weight-loss rate (T_max_), and the residual mass at 700 °C were recorded (Figure 4). According to the TGA results, all samples exhibited a distinct onset of thermal decomposition at approximately 300 °C, attributed to the concurrent degradation of the acrylate matrix constituents (Bis-EMA and TMPTMA) along with PDMS-MMA segments. The onset decomposition temperature (T_5_%) of the PDMS-free control (CP0) was approximately 320.5 °C. With increasing PDMS-MMA concentration, both T_5_% and T_max_ gradually increased, indicating enhanced thermal stability of the composite resin. For example, the CP150 formulation (15 wt% PDMS-MMA) exhibited a T_5_% of 337.2 °C and a T_max_ of 444.3 °C, demonstrating the greatest thermal decomposition resistance among all tested samples.

This improvement in thermal stability can be attributed to the intrinsic characteristics of the siloxane (Si–O–Si) bonds within the PDMS polymer chains. Compared with typical carbon–carbon or carbon–oxygen organic bonds, siloxane bonds exhibit significantly higher bond dissociation energy and superior thermal stability, thereby enhancing the overall heat resistance and structural integrity of the composite resin. Furthermore, due to the inorganic nature of PDMS, thermally stable silica-based residues remain following decomposition, leading to an increase in residual mass at 700 °C with rising PDMS-MMA content. For instance, while the residual mass of CP0 was 6.3%, that of CP150 increased to 18.2%, showing more than a threefold increase.

In summary, PDMS-MMA effectively enhances the thermal stability of photo-curable acrylate resins, imparting favorable characteristics for high-temperature dimensional stability and resistance to thermal decomposition. However, excessive PDMS-MMA concentration may negatively influence optical transparency and potentially affect the photo-curing behavior of the resin system, highlighting the importance of optimizing the formulation composition.

### 3.4. Transmittance Properties of PDMS-MMA Resins

Optical transmittance is a critical property for evaluating the optical clarity, material homogeneity, and overall functionality of materials employed in SLA-based 3D printing. This property is particularly essential for materials intended for micro-opto-electro-mechanical systems (MOEMSs) or optical component fabrication, where high transparency and minimal light scattering are fundamental prerequisites.

**Figure 5:**
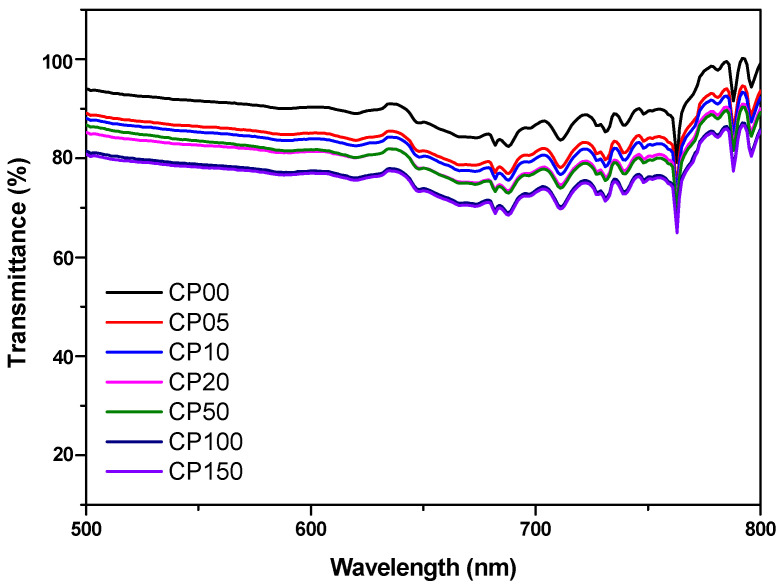
Light transmittance of different PDMS-MMA resins. (Sample thickness is 4.0 mm).

In this study, planar specimens (4.0 mm thick) were fabricated using resins containing various amounts of PDMS-MMA, and their optical transmittance was measured over the 500–800 nm wavelength range using a UV–Vis spectrophotometer (Table 3; Figure 5). The experimental results revealed a monotonic decline in optical transmittance with increasing PDMS-MMA concentration, indicating a direct correlation between PDMS-MMA loading and light attenuation.

The pristine resin without PDMS-MMA (CP0) exhibited a remarkably high transmittance of 93.7%. However, upon the addition of PDMS-MMA at concentrations of 5 wt% or higher, a progressive decrease in transmittance was observed across the visible spectrum. For instance, the CP150 sample (15 wt% PDMS-MMA) showed a reduced transmittance of 81.3%, corresponding to a notable decline in optical clarity and increased light scattering (Table 3).

The observed decrease in optical transmittance may be associated with increased light scattering arising from microstructural heterogeneity within the resin system. Furthermore, differences in refractive index between PDMS-MMA segments and the surrounding acrylate matrix may also contribute to increased light scattering within the cured resin.

In conclusion, while PDMS-MMA incorporation significantly improves mechanical flexibility and thermal stability, its adverse impact on optical clarity must be carefully controlled through formulation optimization. For optically demanding applications, such as microfluidic or photonic devices, precise control of PDMS-MMA concentration is essential to achieve an optimal balance between mechanical performance and optical transparency.

It should be noted that the SLA printing system used in this study operates at a wavelength of 405 nm, which corresponds to the absorption region of the photoinitiator diphenyl (2,4,6-trimethylbenzoyl) phosphine oxide (TPO). At this wavelength, strong absorption by the photoinitiator is required to effectively initiate the photopolymerization reaction. Consequently, the optical transmittance near 405 nm is inherently lower than that in the visible region.

Although the present optical characterization was conducted primarily in the 500–800 nm range to evaluate the transparency of the cured materials, the successful fabrication of complex structures using the SLA system indicates that the resin formulations exhibit a sufficient curing response under 405 nm irradiation. The curing depth and printing fidelity are therefore governed by the balance between photoinitiator absorption and light penetration within the resin.

### 3.5. Shrinkage Properties of PDMS-MMA Resins

**Figure 6:**
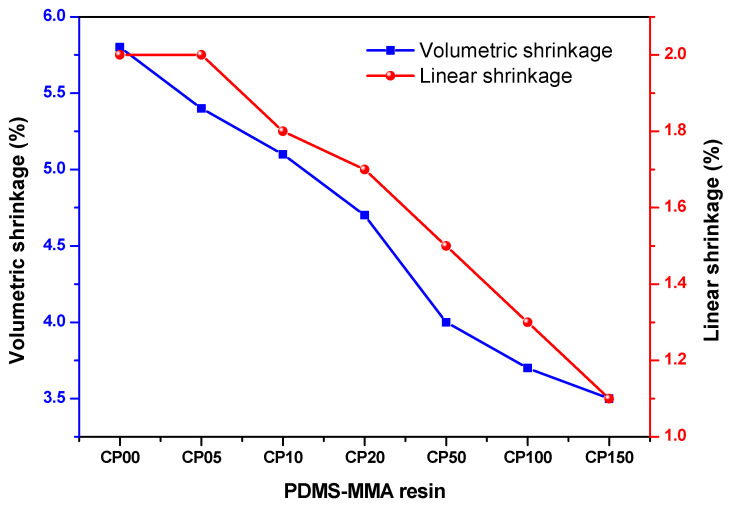
Effect of PDMS-MMA content on the shrinkage of 3D photosensitive materials. Error bars represent standard deviation (*n* = 3).

Curing shrinkage denotes the volumetric contraction that occurs as monomeric species polymerize into crosslinked networks during photo or thermal-induced polymerization processes. This phenomenon often results in dimensional inaccuracy, structural distortion, and compromised mechanical integrity, thereby making it a critical parameter in additive manufacturing technologies, particularly in stereolithography (SLA). In acrylate-based resins, polymerization involves the conversion of carbon–carbon double bonds (C=C) into single bonds, which reduces intermolecular spacing and consequently induces volumetric contraction. Moreover, higher crosslinking density intensifies polymer network contraction, typically leading to volumetric shrinkage between 3% and 10%, depending on the specific resin formulation (Figure 6). Such shrinkage poses major challenges for achieving dimensional fidelity in high-precision additive manufacturing processes.

Unlike conventional non-reactive silicone additives, the PDMS-MMA used in this study is α,ω-dimethacrylate-terminated and therefore contains reactive carbon–carbon double bonds (C=C) at both chain ends. These terminal methacrylate groups can participate in the free-radical photopolymerization reaction together with the acrylate monomers during UV curing, allowing PDMS segments to be incorporated into the crosslinked polymer network. In addition, PDMS-MMA possesses a flexible Si–O backbone and relatively low intermolecular interactions compared with conventional acrylate monomers. As a result, the incorporation of PDMS-MMA can help relieve internal polymerization stress during curing and thereby contribute to a reduction in curing shrinkage of the resin system.

However, due to the polarity difference between hydrophobic PDMS segments and the relatively polar acrylate matrix, partial incompatibility may arise within the resin system. To mitigate this effect, an anionic surfactant (SDS) was introduced to improve dispersion stability and interfacial compatibility of PDMS-MMA within the resin formulation [38]. At elevated PDMS-MMA loadings, increased microstructural heterogeneity within the resin system may contribute to changes in optical transparency of the cured materials.

Furthermore, excessive incorporation of PDMS-MMA may influence the crosslinked network structure of the cured resin. Therefore, an appropriate PDMS-MMA concentration is required to balance curing shrinkage reduction with the overall structural performance of the resin system.

### 3.6. Structural Printing Test

**Figure 7:**
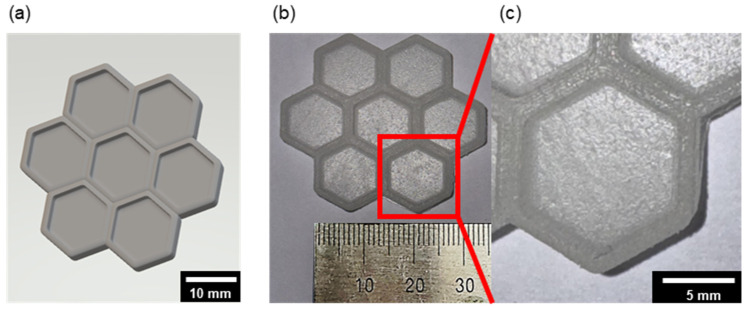
Design and fabrication of the honeycomb structure using SLA printing. (**a**) 3D computer-aided design model of the hexagonal honeycomb structure, (**b**) printed structure using a resin containing 15 wt% PDMS-MMA, and (**c**) magnified view highlighting the surface texture and layer stacking of the printed structure.

To assess the practical printability of the developed photo-curable resin system, a series of experimental fabrication trials was performed using an SLA-based 3D printer. A hexagonal honeycomb structure incorporating internal walls was designed and fabricated to examine key processing parameters, including resin viscosity, curing shrinkage, and layer-stacking uniformity (Figure 7). This geometrically simple two-dimensional structure served as an effective model for assessing surface fidelity and interlayer adhesion quality.

Based on prior results indicating that low to moderate PDMS-MMA resins (≤15 wt%) exhibited reliable printability under standard SLA conditions, only the formulation with the highest PDMS-MMA concentration (15 wt%) was chosen for subsequent printing evaluations. This high-PDMS-MMA formulation was expected to present significant processing challenges owing to its elevated viscosity and diminished optical transmittance and was thus employed to evaluate the printability threshold of the resin system.

**Figure 8:**
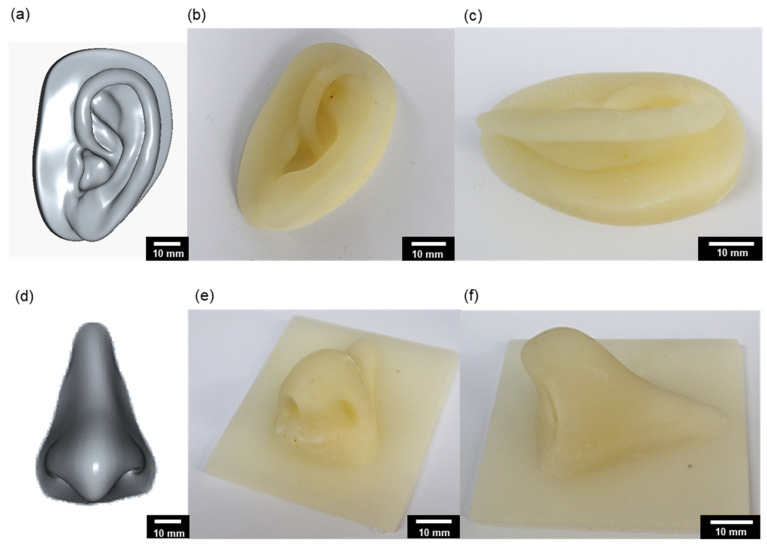
Design and fabrication of ear and nose structures using SLA printing. (**a**) 3D computer-aided design models of the ear structures; (**b**) printed ear model using a resin containing 15 wt% PDMS-MMA; (**c**) side view of the printed ear; (**d**) 3D computer-aided design models of the nose structures; (**e**) printed nose model using a resin containing 15 wt% PDMS-MMA; (**f**) side view of the nose structure.

As an additional validation of the resin’s applicability to complex geometries, 3D anatomical computer-aided design models of the human ear and nose were designed and fabricated using SLA-based 3D printing (Figure 8). These models featured asymmetric geometries and intricately curved surfaces yet were successfully fabricated without any signs of delamination, layer misalignment, or structural deformation. The results demonstrated excellent shape fidelity and surface smoothness, closely resembling the dimensional precision and esthetic quality of real-world prototypes.

Overall, these findings indicate that even at elevated PDMS-MMA concentrations, the resin retains adequate viscosity control and effectively suppresses curing shrinkage, thereby facilitating stable and reliable SLA processing. Moreover, the successful fabrication of complex biomimetic geometries underscores the resin’s broad potential for medical prototyping, bioinspired device engineering, and advanced soft-material manufacturing.

## 4. Conclusions

In this study, photo-curable acrylate resins were formulated by incorporating different concentrations of PDMS-MMA, and their physicochemical properties were systematically characterized and evaluated. The results demonstrated that PDMS-MMA incorporation moderately increased resin viscosity but effectively reduced curing shrinkage, thereby improving dimensional accuracy and overall printing stability during SLA-based 3D printing.

From a mechanical perspective, moderate incorporation of PDMS-MMA improved both tensile strength and elongation at break, with the best mechanical performance observed at 5 wt% PDMS-MMA. However, excessive PDMS-MMA loading resulted in a decrease in tensile strength, indicating that an optimal PDMS-MMA concentration is required to balance flexibility and structural integrity.

A structural printing test using a hexagonal honeycomb model confirmed that the developed resin system exhibited excellent reproducibility, dimensional accuracy, and surface fidelity under SLA-based 3D printing conditions.

Overall, this study provides fundamental insights into the design, formulation, and functional applicability of PDMS-MMA-modified photo-curable composite resins. The findings are expected to serve as a valuable reference for future developments in optical component fabrication, flexible device engineering, and biomedical 3D printing.

## Figures and Tables

**Table 1:** Formulation compositions of the PDMS-MMA modified photo-curable resins (wt%).

Specimen	Composition (wt%)		
	Photopolymer Base	PDMS-MMA	Sodium Dodecyl Sulfate
CP0	100	0	0
CP05	99.5	0.5	0.1
CP10	99.0	1.0	0.1
CP20	98.0	2.0	0.1
CP50	95.0	5.0	0.3
CP100	90.0	10.0	1.0
CP150	85.0	15.0	1.5

**Table 2:** Viscosity and mechanical properties of PDMS-MMA resin formulations. Values are presented as mean ± standard deviation (*n* = 3).

Specimen	Viscosity (cP)	Tensile Strength (MPa)	Elongation at Break (%)
CP0	332 ± 5.8	3.56 ± 0.05	28.4 ± 0.10
CP05	345 ± 12.0	3.59 ± 0.09	47.6 ± 1.00
CP10	372 ± 10.4	3.68 ± 0.07	63.2 ± 1.87
CP20	391 ± 8.3	4.16 ± 0.10	85.0 ± 0.50
CP50	423 ± 5.4	5.95 ± 0.09	103.6 ± 0.91
CP100	489 ± 7.2	4.28 ± 0.10	99.4 ± 1.12
CP150	530 ± 4.5	2.51 ± 0.25	95.8 ± 2.75

**Table 3:** Effect of PDMS content on transmittance and curing shrinkage of resins. Values are presented as mean ± standard deviation (*n* = 3).

Name	Transmittance (%) (500 nm)	Shrinkage (%)	
		Volumetric Shrinkage	Linear Shrinkage
CP0	93.7 ± 1.0	5.8 ± 0.02	2.0 ± 0.01
CP05	89.3 ± 1.1	5.4 ± 0.02	2.0 ± 0.01
CP10	88.2 ± 1.3	5.1 ± 0.03	1.8 ± 0.02
CP20	85.4 ± 1.1	4.7 ± 0.02	1.7 ± 0.02
CP50	87.1 ± 0.9	4.0 ± 0.02	1.5 ± 0.01
CP100	82.8 ± 0.2	3.7 ± 0.03	1.3 ± 0.01
CP150	81.3 ± 1.1	3.5 ± 0.01	1.1 ± 0.02

## Data Availability

The original contributions presented in this study are included in the article. Further inquiries can be directed to the corresponding author.
